# Elderly people with human T-cell leukemia virus type 1-associated myelopathy present an early impairment in cognitive skills

**DOI:** 10.1055/s-0043-1763486

**Published:** 2023-04-14

**Authors:** Beatriz Rezende Matos de Sousa, Ludimila Labanca, Maria Luiza Diniz, Nathália de Castro Botini Rausse, Denise Utsch Gonçalves

**Affiliations:** 1 Universidade Federal de Minas Gerais, Faculdade de Medicina, Programa de Pós-Graduação em Infectologia e Medicina Tropical, Belo Horizonte MG, Brazil.; 2 Universidade Federal de Minas Gerais, Faculdade de Medicina, Programa de Pós-Graduacão em Ciências Fonoaudiológicas, Belo Horizonte MG, Brazil.; 3 Universidade Federal de Minas Gerais, Faculdade de Medicina, Departamento de Fonoaudiologia, Belo Horizonte MG, Brazil.; 4 Universidade Federal de Minas Gerais, Faculdade de Medicina, Departamento de Otorrinolaringologia, Belo Horizonte MG, Brazil.

**Keywords:** Aged, Human T-lymphotropic Virus 1, Paraparesis, Tropical Spastic, Event-Related Potentials, P300, Neuropsychological Tests, Cognition, Idoso, Vírus Linfotrópico T Tipo 1 Humano, Paraparesia Espástica Tropical, Potenciais Evocados P300, Testes Neuropsicológicos, Cognição

## Abstract

**Background** Cerebral changes occur in individuals with human T-cell leukemia virus type 1 (HTLV-1)-associated myelopathy (HAM) and seem to predominate in subcortical areas. Little is known about the cognitive decline in the elderly living with HTLV-1.

**Objective** To evaluate the cognitive aging of individuals infected with HTLV-1 aged ≥ 50 years.

**Methods** This is a cross-sectional study of former blood donors infected with HTLV-1 who have been followed in the cohort of the Interdisciplinary Research Group on HTLV-1 since 1997. The groups of study consisted of 79 HTLV-1 infected individuals aged ≥ 50 years, with 41 of them presenting symptomatic HAM and 38 being asymptomatic carriers, and 59 seronegative individuals (controls) aged ≥ 60 years. All were submitted to the P300 electrophysiological test and neuropsychological tests.

**Results** Individuals with HAM presented delayed P300 latency in relation to the other groups, and this latency delay increased progressively with aging. The performance of this group in the neuropsychological tests was also the worst. The HTLV-1- asymptomatic group performance was similar to that of the control group.

**Conclusions** Individuals with HAM presented cognitive decline that progressed with aging and, although HTLV-1-asymptomatic carriers appear to present cognitive aging similar to that of healthy elderly people, concern about a subclinical cognitive impairment is warranted in this population.

## Resumo

**Antecedentes** Alterações cerebrais ocorrem em indivíduos com mielopatia associada ao vírus da leucemia de células T humanas tipo 1 (HTLV-1) (HAM) e parecem predominar em áreas subcorticais. Pouco se sabe sobre o declínio cognitivo em idosos vivendo com HTLV-1.

**Objetivo** Avaliar o envelhecimento cognitivo de indivíduos infectados pelo HTLV-1 com idade ≥ 50 anos.

**Métodos** Trata-se de um estudo transversal com ex-doadores de sangue infectados pelo HTLV-1 acompanhados na coorte do Grupo Interdisciplinar de Pesquisa em HTLV-1 há 20 anos. Os grupos de estudo foram compostos por 79 indivíduos infectados pelo HTLV-1 com idade ≥ 50 anos, sendo que 41 apresentavam HAM e 38 eram portadores assintomáticos, e 59 indivíduos soronegativos (controles) com idade ≥ 60 anos. Todos foram submetidos ao teste eletrofisiológico P300 e testes neuropsicológicos.

**Resultados** Indivíduos com HAM apresentaram atraso na latência do P300 em relação aos demais grupos, e esse atraso de latência aumentou progressivamente com o envelhecimento. O desempenho desse grupo nos testes neuropsicológicos também foi o pior. O desempenho do grupo HTLV-1- assintomático foi semelhante ao do grupo controle.

**Conclusão** Indivíduos com HAM apresentaram declínio cognitivo que progrediu com o envelhecimento e, embora os portadores assintomáticos do HTLV-1 pareçam apresentar envelhecimento cognitivo semelhante ao dos idosos saudáveis, justifica-se a preocupação com um comprometimento cognitivo subclínico nessa população.

## INTRODUCTION

The human T-cell leukemia virus type 1 (HTLV-1) is endemic in some regions of the world, and Japan, Africa, the Caribbean islands, and South America are the areas of greatest circulation of this virus. In these areas, the prevalence gradually increases with age, especially among women. 1 2 The prevalence analysis of HTLV-1 infection in Brazil's blood bank in 2016 indicates that every 1,008 in 100,000 blood-donor candidates have HTLV-1 infection, and 354 (35%) are older than 50-year-old. 2 Thus, a higher prevalence of HTLV-1 infection is expected in the elderly population compared with the younger.

Human T-cell leukemia virus type 1 can cause HTLV-1-associated myelopathy (HAM), a neurological disease in which diffuse loss of myelin and axons occurs in the medulla, especially in the thoraco-lumbar region. 3 The spinal cord damage leads to changes in motor and autonomic function that impair functionality and activities of daily living. Bladder dysfunction, constipation, and gait changes are the most common alterations. 4 Consequently, the studies about the neurological manifestations associated with HTLV-1 have been focused on the spinal cord disease and, comparatively, fewer studies have considered the brain changes associated with HTLV-1 infection, although brain changes in the asymptomatic phase of the infection have already been found. 5 In HAM, the magnetic resonance imaging (MRI) showed lesions similar to the ones found in the human immunodeficiency virus (HIV)-associated neurocognitive disorders (HANDs). 6 Therefore, cognitive impairment can be a neurological manifestation associated with HTLV-1 infection. 7 8 9 10 11

The literature is scarce regarding the cognitive performance of HTLV-1-infected people over 50 years of age, and, then, we aimed at evaluating the performance of people living with HTLV-1 over 50 years of age in terms of attention, memory, general cognitive ability, executive functions, motor and psychomotor speed, and manual dexterity. Our hypothesis is that HTLV-1-infected people, especially those who develop HAM, present cognitive impairment parallel to the spinal cord damage.

## METHODS

### Ethical aspects

This research was conducted in accordance with the principles expressed in the Declaration of Helsinki and was approved by the research ethics committee from Universidade Federal de Minas Gerais (COEP UFMG), logged under protocol CAAE 92928518.3.0000.5149 and number 2898825. All participants provided voluntary written consent and declared that they were aware of the study procedures and their choice to participate.

### Participants

All participants in this study take part in the cohort of the Interdisciplinary Research Group on HTLV-1 (GIPH). They are seronegative blood donors and former blood donors infected with HTLV-1 from the state of Minas Gerais, Brazil, who have been followed up since 1997. In this study, 138 individuals from this cohort were included and distributed into the groups with and without HTLV-1 infection.

The study groups consisted of 59 HTLV-1-seronegative blood donors (controls), as assessed by enzyme-linked immune sorbent assay (ELISA), and 79 HTLV-1-infected ex-blood donors, diagnosed by ELISA and confirmed by Western blot analysis (WB HTLV 2.4, Genelabs Diagnostics, Singapore Science Park 1, Singapore) or by real-time polymerase chain reaction (RT-PCR). The HTLV-1 group was divided into 41 HAM and 38 HTLV-1-asymptomatic carriers, according to the neurological assessment. 12 The score on the Osame motor disability score (OMDS) scale was ≥ 1 for individuals with HAM and 0 for HTLV-1-asymptomatic carriers. 13

### Study design

A cross-sectional, comparative study was carried out, nested to an open cohort, in which HTLV-1 seropositive adults ≥ 50 years of age were compared to HTLV-1 seronegative adults ≥ 60 years of age in relation to cognitive abilities that were assessed using the P300 electrophysiological test and neuropsychological tests. The decision to evaluate individuals infected with HTLV-1 who were 10 years younger than the seronegative controls was based on the comparison of the aging of these patients with the pattern of premature aging that occurs in HIV infection, in which individuals above 50 years of age are classified as elderly people. 14 Human T-cell leukemia virus type 1 and HIV cause structural brain changes seen in MRI that seem to be similar. 6

The individuals recruited for this research were initially submitted to a clinical and neurological evaluation and, later, to an audiological evaluation that consisted of tonal and vocal audiometry, imitanciometry with 226 Hz probe (Interacoustics, Middelfart, Denmark), and brainstem auditory evoked potential (BAEP) at 90 dB hearing level (dB HL) (model MASBE/ATCPlus, Contronic Ltda., Pelotas, RS, Brazil). Finally, the participants included in this study were submitted to cognitive assessment.

The exclusion criteria were coinfection with HIV and/or HTLV-2, patients with diagnoses of dementia, alcoholism, or drug addiction, mental illness, sequelae due to opportunistic infections in the central nervous system (CNS), hearing loss greater than 40 dB HL at frequencies from 500 Hz to 4,000 Hz, any alteration in the middle ear or deformities in the external auditory canal, alteration of the auditory pathways to the brainstem, and patients with depressive symptoms scored above 5 on the geriatric depression scale of 15 items. 15

### Neurocognitive tests

#### Electrophysiological test – Auditory P300 Test

The event-related potential (ERP) is an electrophysiological method that allows the capture of human neuroelectric activities when the individual is subjected to a specific event, such as an auditory stimulus. The P300 complex of the ERP consists of a large positive waveform whose peak occurs around 300 milliseconds (ms) after the stimulus presentation. The P300 is generated by a complex neural network, in which connections occur involving the thalamus, temporal-parietal cortex, prefrontal cortex, hippocampus and limbic region, being closely related to short-term memory and attention. 16 The P300 latency increases with aging, and latency prolongation beyond the accepted limits considered as normal for a determined age group indicates the occurrence of a cognitive dysfunction. 16 17 18 Sleep deprivation can interfere with the generation of P300. 19 So, the participants were instructed to sleep at least 6 hours on the night before the auditory P300 test. Drugs that act on the CNS, such as benzodiazepines, neuroleptics, and anticonvulsants, can delay P300. So, these drugs were controlled in the statistical analysis.

The auditory P300 test was performed with the individual in a seated and comfortable position, with their eyes open, in an electric and acoustically treated room with dim light, using the MASBE/ACTPlus equipment (Contronic Ltda. The participants' skin was cleaned with abrasive paste, and the electrodes were fixed according to the international electrode system (IES) 10-20 standard, with the negative electrodes placed on the left (A1) and right (A2) earlobes, the ground electrode on the forehead (Fp1), and the active electrode on the forehead (Fz). 20 Studies have already shown that obtaining P300 latency is not affected by the location chosen for fixing the active electrode, whether in Fz, Cz or Pz. 7 21 22 In relation to the electrode impedance, the maximum accepted value for each electrode was 3 kΩ, and the difference in impedance between the electrodes was, at most, 1 kΩ. The stimulus used was the tone burst with binaural presentation with TDH-39 phone (Huntington, NY, United States) and intensity of 90 dB HL. For the generation of P300, the auditory *oddball* paradigm was used, in which 2 stimuli were presented randomly, with one of them occurring frequently and the other in a rare way (target). The individuals were instructed to identify and mentally count the number of rare stimuli (target). The parameters defined for the stimulus characterized as frequent were a tone burst sound in the frequency of 1,000 Hz, 50 cycles of duration, 20% rise and decay time, 60% plateau, trapezoidal envelope, and alternating polarity. For the tone burst stimulus characterized as rare, the parameters were a frequency of 2,000 Hz, 100 cycles of duration, 20% rise and decay time, 60% plateau, trapezoidal envelop and alternating polarity. For each exam, 300 stimuli were presented, with a rate of 0.8 stimuli per second, with 80% of frequent stimuli and 20% rare. The amplifiers were configured with the 200 µV full scale, the 01 Hz high-pass filter, the 20 Hz low-pass filter and the 60 Hz notch filter. The time window was 1,000 ms and the electroencephalogram was enlarged 50,000 times. Before starting the exam, the stimuli were presented so that the participants became familiar with the test. The procedure was repeated once more to guarantee the replication of the tracing. P300 analysis was performed separately by two independent examiners who were experienced in electrophysiological examinations. The analysis was masked in relation to the group.

The P300 component is the largest positive peak between 250 and 500 ms following the presentation of the stimulus, and it occurs after the N100, P160, and N200 components. The study of the P300 can regard its amplitude or latency. Amplitude can be interpreted as the number of cognitive resources that an individual allocates in a cognitive task and, therefore, refers to mental effort. 16 Latency, on the other hand, allows inference regarding performance in specific cognitive skills, especially attention and working memory. Thus, the P300 latency (P3b) is more commonly used to diagnose and monitor cognitive impairment and, for this reason, was the variable considered in the present research. 23

#### Neuropsychological tests

After being submitted to the auditory P300 test, the participants underwent neuropsychological tests to assess specific cognitive domains. The tests applied were Raven colored progressive matrices (RAVEN), which assesses general cognitive ability 24 ; Rey auditory-verbal learning test (RAVLT) adapted for the Brazilian elderly population, which evaluates recent memory, verbal learning, susceptibility to interference, retention of information after a certain period of time in which other activities are performed, and memory recognition 25 ; frontal assessment battery (FAB), which evaluates executive functions 26 ; international HIV dementia scale (IHDS), which identifies individuals at risk of developing HIV-associated neurocognitive disorders (HANDs), 27 and Nine-hole peg test (NHPT) dominant hand (DH) and non-dominant hand (NDH), which assesses manual dexterity. 28 Higher score as a result on the NHPT indicates worse performance because it shows that the individual spent more time to complete the test. Lower scores for the other tests indicate poorer performance.

### Statistical analysis

The measurement of interrater agreement of the P300 result was applied using the intraclass correlation coefficient (ICC), in which a value ≥ 0.70 indicates good reliability. 29 Descriptive analysis was performed using measures of central tendency and variability for continuous variables and analysis of frequency for categorical variables. The pattern of distribution of the continuous variables was performed using the Shapiro-Wilk and Kolmogorov-Smirnov tests. The comparison of continuous variables between groups was performed using the analysis of variance (ANOVA) with Bonferroni, Kruskal-Wallis, and Mann-Whitney tests. Spearman correlation coefficients were calculated to find correlation between variables. To compare categorical variables between groups, the Chi-squared test was used. For statistical significance, an alpha error of 0.05 was considered. The analyses were performed using IBM SPSS Statistics for Windows, version 20.0 (IBM Corp., Armonk, NY, USA).

## RESULTS

### Characteristics of the participants

The groups were similar regarding educational level ( *p* = 0.224) and gender ( *p* = 0.895). As expected, the average age of the control group was higher than the one of the HTLV-1-infected groups ( *p* ˂ 0.001). These data are summarized in Table 1 .

**Table 1:** General characteristics of the asymptomatic and HTLV-1-infected groups (n = 138)

Variables	Seronegative	HTLV-1-asymptomatic	HAM	*p* -value	
**(n = 59)**	**(n = 38)**	**(n = 41)**
**Age (years old)**	66 ± 3.6	61 ± 6.8	61 ± 8.3	**< 0.001** ^a^	**0.001 (G1 > G2)** ^a^
					**0.002 (G1 > G3)** ^a^
**Education (years of schooling)**	5 (4/10)	7 (4/10)	5 (3/8)	0.224 ^b^	−
**Female**	40 [68]	24 [63]	27 [66]	0.895 ^c^	−
						**OMDS**	0	0	4 (1/5)	**< 0.001** ^b^	**0.001 (G1 > G3)** ^b^
**0.001 (G2 > G3)** ^b^										

Abbreviations: G1, seronegative group; G2, HTLV-1-asymptomatic group; G3, HAM group; HAM, HTLV-1 associated myelopathy; HTLV-1, human T-cell lymphotropic virus type 1; OMDS, Osame motor disability score; *p* , significance probability.

Notes: Data are expressed as mean ± standard deviation for continuous variables with normal distribution, median (Quartil Q1/Quartil Q3) for continuous variables with non-normal distribution or absolute numbers [percentage] for categorical variables. ^a^ ANOVA test – Bonferroni; ^b^ Kruskal-Wallis test; ^c^ Chi-Square test.

Some patients used medications with possible potential deleterious effects on the cognition: 7 (5%) clonazepam, 4 (3%) baclofen, 3 (2%), gabapentin, and less than 1% reported oxcarbazepine, carbamazepine, pregabaline, or phenobarbital. No association was found between cognitive performance and the use of these medications.

### Electrophysiological test – Auditory P300 Test

**Figure 1:**
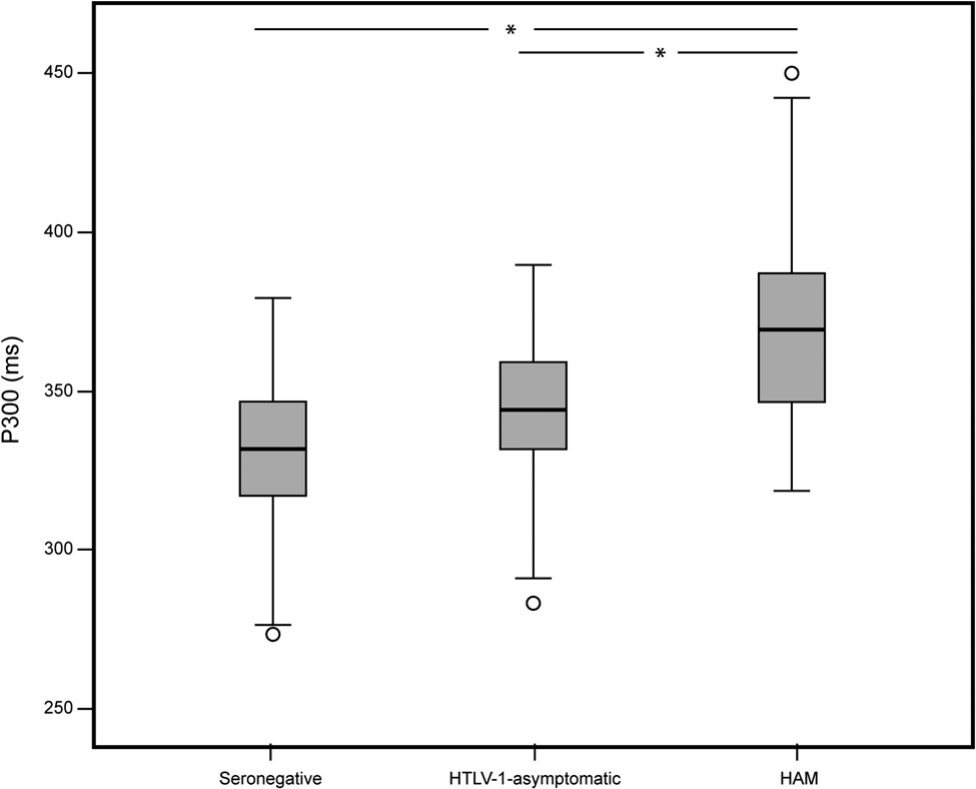
Between-groups comparison of P300 latency obtained from the seronegative group (n = 59), human T-cell lymphotropic virus type 1 (HTLV-1)-asymptomatic group (n = 38) and HTLV-1 associated myelopathy (HAM) group (n = 41). Note: The boxes denote the P300 latency in each of the three groups. The horizontal line in the box represents the median; the box hinges represent the first (Q1) and third quartiles (Q3). Upper and lower whiskers extend from the hinge to the highest/lowest value that lies within the 1.5 interquartile range (IQR) of the hinge.Notes: * *p* < 0.001; *p* , significance probability (independent-samples Kruskal-Wallis test for specific comparisons).

In the P300 interrater analysis, a good reliability was found in the double masking analysis of the P300 latency (ICC = 0.984, *p* < 0.001). So, the P300 measures were shown to be adequate. The P300 latency was delayed in the HAM group when compared with the HTLV-1-asymptomatic and control groups ( Figure 1 ).

The difference was clarified in the stratified analysis by age group. Comparisons were made in groups and between groups for the ranges of 50 to 59, 60 to 69, and 70 to 79 years of age. The HAM group had the most delayed latency compared with the HTLV-1-asymptomatic and control groups ( Table 2 ).

**Table 2:** Comparison of P300 latency stratified by the age ranges 50 to 59, 60 to 69, and 70 to 79 in the asymptomatic and HTLV-1-infected groups and between them

P300 latency (ms)									
	G1 (seronegative) - n = 59		G2 (HTLV-1-asymptomatic) - n = 38			G3 (HAM) - n = 41			*p* -value
**General comparison**	331.33 (316.19/346.47)		343.95 (329.44/359.02)			−			0.142
	331.33 (316.19/346.47)		−			369.18 (346.61/386.84)			**< 0.001** ^a^
	−		343.95 (329.44/359.02)			369.18 (346.61/386.84)			**< 0.001** ^a^
**Comparison by age group**	**[60-69]**	**[70-79]**	**[50-59]**	**[60-69]**	**[70-79]**	**[50-59]**	**[60-69]**	**[70-79]**	P **-Value**
	**n = 46**	**n = 13**	**n = 18**	**n = 13**	**n = 7**	**n = 18**	**n = 13**	**n = 10**	
**G1 [60-69] x**	332.59	330.92	−	−	−	−	−	−	0.862
**G1 [70-79]**	(317.14/346.47)	(314.93/345.21)							
**G2 [50-59] x**	−	−	345.21	343.95	−	−	−	−	0.795
**G2 [60-69]**			(329.44/357.19)	(314.93/361.56)					
**G2 [50-59] x**	−	−	345.21	−	344.00	−	−	−	0.739
**G2 [70-79]**			(329.44/357.19)		(336.00/364.13)				
**G2 [60-69] x**	−	−	−	343.95	344.00	−	−	−	0.968
**G2 [70-79]**				(314.93/361.56)	(336.00/364.13)				
**G3 [50-59] x**	−	−	−	−	−	365.65	366.65	−	0.298
**G3 [60-69]**						(340.79/377.86)	(348.99/389.36)		
**G3 [50-59] x**	−	−	−	−	−	365.65	−	398.19	**0.003** ^b^
**G3 [70-79]**						(340.79/377.86)		(378.64/419.64)	
**G3 [60-69] x**	−	−	−	−	−	−	366.65	398.19	**0.040** ^b^
**G3 [70-79]**							(348.99/389.36)	(378.64/419.64)	
**G1 [60-69] x**	332.59	−	345.21	−	−	−	−	−	0.099
**G2 [50-59]**	(317.14/346.47)		(329.44/357.19)						
**G1 [60-69] x**	332.59	−	−	343.95	−	−	−	−	0.167
**G2 [60-69]**	(317.14/346.47)			(314.93/361.56)					
**G1 [70-79] x**	−	330.92	345.21	−	−	−	−	−	0.128
**G2 [50-59]**		(314.93/345.21)	(329.44/357.19)						
**G1 [70-79] x**	−	330.92	−	343.95	−	−	−	−	0.227
**G2 [60-69]**		(314.93/345.21)		(314.93/361.56)					
**G1 [70-79] x**	−	330.92	−	−	344.00	−	−	−	0.191
**G2 [70-79]**		(314.93/345.21)			(336.00/364.13)				
**G1 [60-69] x**	332.59	−	−	−	−	365.65	−	−	**< 0.001** ^b^
**G3 [50-59]**	(317.14/346.47)					(340.79/377.86)			
**G1 [60-69] x**	332.59	−	−	−	−	−	366.65	−	**< 0.001** ^b^
**G3 [60-69]**	(317.14/346.47)						(348.99/389.36)		
**G1 [70-79] x**		330.92				365.65			**0.005** ^b^
**G3 [50-59]**		(314.93/345.21)				(340.79/377.86)			
**G1 [70-79] x**	−	330.92	−	−	−	−	366.65	−	**0.002** ^b^
**G3 [60-69]**		(314.93/345.21)					(348.99/389.36)		
**G1 [70-79] x**	−	330.92	−	−	−	−	−	398.19	**< 0.001** ^b^
**G3 [70-79]**		(314.93/345.21)						(378.64/419.64)	
**G2 [50-59] x**	−	−	345.21	−	−	365.65	−	−	**0.025** ^b^
**G3 [50-59]**			(329.44/357.19)			(340.79/377.86)			
**G2 [60-69] x**	−	−	−	343.95	−	−	366.65	−	**0.024** ^b^
**G3 [60-69]**				(314.93/361.56)			(348.99/389.36)		
**G2 [70-79] x**	−	−	−	−	344.00	−	−	398.19	**0.008** ^b^
**G3 [70-79]**					(336.00/364.13)			(378.64/419.64)	
**G2 [60-69] x**	−	−	−	343.95	−	365.65	−	−	0.072
**G3 [50-59]**				(314.93/361.56)		(340.79/377.86)			
**G2 [70-79] x**	−	−	−	−	344.00	365.65	−	−	0.146
**G3 [50-59]**					(336.00/364.13)	(340.79/377.86)			
**G2 [70-79] x**	−	−	−	−	344.00	−	366.65	−	0.103
**G3 [60-69]**					(336.00/364.13)		(348.99/389.36)		

Abbreviations: G1, Seronegative group; G2, HTLV-1-asymptomatic group; G3, HAM group; HAM, HTLV-1 associated myelopathy; HTLV-1, human T-cell lymphotropic virus type 1; ms, milliseconds; p, significance probability.

Notes: Data are expressed as median (Q1 quartile/Q3 quartile). ^a^ Kruskal-Wallis test; ^b^ Mann-Whitney test.

**Figure 2:**
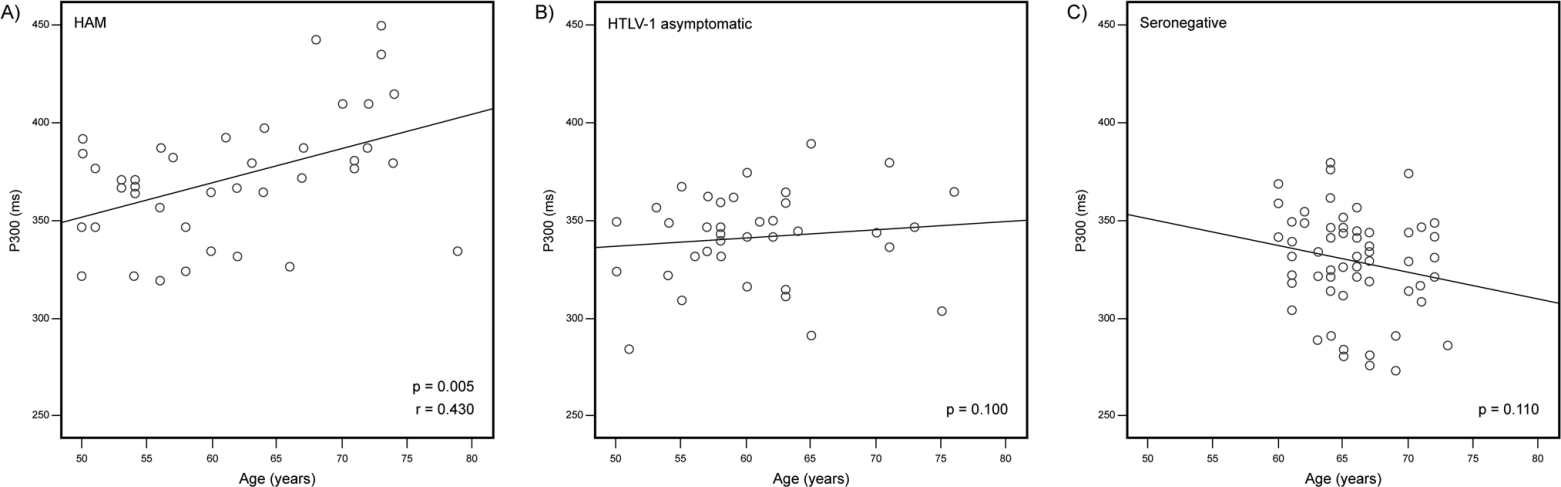
Scatter graph showing the correlation between P300 latency and the aging of (A) individuals with HTLV-1-associated myelopathy (HAM), (B) HTLV-1-asymptomatic carriers, and (C) seronegative controls distributed by age. Note: A positive correlation between increasing age and prolongation of P300 latency was detected only for the HAM group.Notes: *p* , significance probability; r, correlation coefficient; Spearman rho test.

A regular and progressive P300 delay was observed from the ranges of 50 to 59, 60 to 69, and 70 to 79 years of age in the HAM group ( Figure 2 and Table 2 ). The HTLV-1-asymptomatic group presented a different P300 pattern. In this group, the latency, although slightly delayed, was considered similar to the one found in the control group for all the age ranges ( Table 2 ). In addition, a stable P300 latency with aging was observed, as it occurred in the control group ( Figure 2 and Table 2 ).

In order to characterize the trend of P300 latency with the aging in the groups with and without the HTLV-1 infection, a correlation between P300 latency and age was performed, and a positive correlation was found between an increasing age and P300 latency prolongation only for the HAM group ( Figure 2 ).

### Neuropsychological tests

The performance of the individuals in neuropsychological tests can be seen in Table 3 . The general cognitive ability (RAVEN) was found to be similar among the groups. The analysis of the RAVLT test, including all the scales, was found to be different in the A5 subscale, which evaluates verbal learning, and in A7, which evaluates verbal recall related to long-term memory, indicating worse performance of the HAM group compared to the control group, but not to the HTLV-1-asymptomatic group. The test of executive functions using FAB and motor skills using IHDS, which are more specific for HAND, found a worse performance in theHAM group, compared with the HTLV-1-asymptomatic and control groups. In addition, in the analysis of the time spent performing the NHPT, the HAM group presented a worse result using either the dominant or non-dominant hand when compared with the other two groups.

**Table 3:** Comparative analysis between the seronegative (n = 59), HTLV-1-asymptomatic carriers (n = 38), and HAM (n = 41) groups regarding performance in neuropsychological tests

Variable		Seronegative	HTLV-1-asymptomatic	HAM	*P* -value	
**General intelligence**	RAVEN(total)	23 (17/27)	25 (19/30)	23 (19/28)	0.275 ^a^	−
**Screening tests**	FAB (total)	17 (14/17)	17 (15/18)	14 (13/16)	**0.003** ^a^	**0.023 (G1 > G3)** ^a^
						**0.003 (G2 > G3)** ^a^
	IHDS (total)	11 (9/12)	11 (9/12)	10 (9/11)	**0.020** ^a^	**0.044 (G1 > G3)** ^a^
						**0.041 (G2 > G3)** ^a^
**Episodic memory**	RAVLT A5 (total)	11 (10/13)	11 (10/13)	9 (8/12)	**0.010** ^a^	**0.008 (G1 > G3)** ^a^
	RAVLT A7 (total)	8 (7/11)	7 (6/10)	7 (5/10)	**0.040** ^a^	**0.046 (G1 > G3)** ^a^
**Motor processing and attention**	NHPT - DH (time - seconds)	20 (19/24)	21 (20/26)	25 (22/28)	**<0.001** ^a^	**< 0.001 (G1 < G3)** ^a^
						**0.019 (G2 < G3)** ^a^
	NHPT - NDH (time - seconds)	22 (20/24)	22 (21/25)	25 (24/30)	**< 0.001** ^a^	**<0.001 (G1 < G3)** ^a^
						**0.006 (G2 < G3)** ^a^

Abbreviations: DH, dominant hand; FAB, frontal assessment battery; G1, seronegative group; G2, HTLV-1-asymptomatic group; G3, HAM group; HAM, HTLV-1 associated myelopathy; HTLV-1, human T-cell lymphotropic virus type 1; IHDS, international HIV dementia scale; NDH, non-dominant hand; NHPT, nine-hole peg test; *p* , significance probability; RAVLT, Rey auditory-verballearning test.

Notes: Data are expressed in median (Q1 quartile/Q3 quartile). ^a^ Kruskal-Wallis test.

## DISCUSSION

The understanding of the cognitive aging process that occurs in individuals living with HTLV-1 can contribute to early therapeutic decisions that favor the maintenance of the cognitive skills. This research discusses HTLV-1 infection and aging. Cognitive changes were more severe in individuals with HAM compared to the findings of the other groups. Human T-cell leukemia virus type 1-asymptomatic carriers presented a performance closer to that observed in the control group, except for the findings in the RAVLT test ( Table 2 and Table 3 ).

The P300 latency was delayed in the HAM group in comparison to the seronegative controls. Even when comparing participants in the older age group among the seronegative controls (70–79) and in the younger age group among those with HAM (50–59), the difference remained ( Table 2 ). In fact, a delayed P300 latency in individuals with HAM has already been noted. 7 Considering that only the HAM group showed a consistent P300 latency prolongation in comparison to the seronegative controls ( Figure1 and Table 2 ) and that this delay was correlated with aging ( Figure 2 ), it was assumed that individuals with HAM presented an early cognitive decline that could not be explained by aging alone, as this same trend did not occur in the HTLV-1-asymptomatic group. The results of the neuropsychological tests support this hypothesis. The HAM group had greater difficulty to perform such tests compared with the other groups, especially the tests that are more related to subcortical changes – FAB, IHDS, and NHPT ( Table 3 ). A study on P300 in elderly people living with HIV, using a similar methodology to that of the present study, observed similar changes in the HAM group to the ones found by us. 30 In fact, the HIV-positive individuals over 50 years of age have an increased risk of cognitive impairment, with changes that are found mainly in attention, memory, fluency and executive functions. 12 These findings reinforce the suspicion that HIV and HTLV-1 may cause lesions in the CNS that are similar in terms of location and pathophysiology. 31 The HTLV-1 virus causes an uncontrolled inflammation in the CNS, leading to a progressive demyelination, which seems to occur in HAND. 32 33 The predominance of an inflammatory profile in the CNS of individuals with HAM is well established. 34

The P300 latency maintained a pattern related to the aging that varied according to the HTLV-1 group. Differently from the results presented by the individuals with HAM, the P300 latency remained stable with the aging in HTLV-1 asymptomatic carriers, similar to what was observed in the control group ( Table 2 and Figure 2 ). A progressive P300 latency delay is expected to occur with aging. 17 35 On the other hand, studies about this subject have been scarce, and aging without a concomitant P300 latency delay has also been published. 30

When a comparison was made between the HAM and HTLV-1 asymptomatic groups, an unexpected P300 latency similarity appeared in the comparison between the HAM youngest age group and the eldest HTLV-1-asymptomatic age group ( Table 2 ). This is consistent with the absence of difference between the HTLV-1-asymptomatic and HAM groups in terms of episodic memory (RAVLT A5 and A7) ( Table 3 ). Possibly, some of the HTLV-1-asymptomatic carriers presented a memory impairment that characterized an intermediate clinical syndrome between the asymptomatic phase and HAM and may have already developed neurological manifestations, both medullary and subcortical, but in a subclinical form. 8 9 11 36 37 38 39 Therefore, in spite of the HTLV-1-asymptomatic group having shown a good performance in most neuropsychological tests, we cannot rule out the possibility of the virus action in the CNS of this population. Some studies have demonstrated that HTLV-1-asymptomatic individuals presented a worse performance in episodic memory and also in other subcortical cognitive skills when compared with controls. 8 In the GIPH cohort, a previous study has demonstrated neurophysiological changes associated with cognitive dysfunction in HAM, which was also found in the asymptomatic phase of the infection. 10

Regarding the predominance of the female gender in the GIPH cohort, which was represented by the predominance of women in the present work, this reflects the epidemiology of HTLV-1 in Brazil and in the world. 1 2 This bias did not interfere in the findings of the present work, since it has already been demonstrated in the elderly population that there is no difference in P300 latency between genders. 17 Medications such as benzodiazepines and anticonvulsants can affect P300 amplitude. 40 In our study, only 10% of patients used at least one of these drugs for symptomatic mitigation, and in the data analysis, no association was found between cognitive performance and the use of these medications.

The limitation of the present study was the selection of neuropsychological tests that focused on subcortical dementia and not on a global cognitive evaluation. The reason was that the studied population included fragile HAM elderly people who would not tolerate a long-time test. So, the time taken to run the entire battery of cognitive tests was a limiting factor. The inclusion of P300 was important because this test is easy to apply, it is not tiring and, finally, changes in the electrophysiological tests precede changes observed in the neuropsychological tests.

In conclusion, HAM was associated with a cognitive decline, possibly of subcortical onset, that seems to worsen with aging. Those infected with HTLV-1, classified as asymptomatic carriers, did not appear to exhibit a cognitive decline that was different from healthy seronegative elderly. However, HTLV-1-asymptomatic individuals with any cognitive complaint, especially regarding memory, should be submitted to a battery of neurocognitive tests in order to investigate cases of possible subclinical manifestation of the disease.
